# Supplementary material for: DNA methylation architecture of the ACE2 gene in nasal cells of children
Source: Sci Rep. 2021 Mar 29;11:7107. doi: 10.1038/s41598-021-86494-7 (PMC8007733; doi:10.1038/s41598-021-86494-7)

**Supplemental Material**

**Title**: DNA Methylation Architecture of the *ACE2* gene in Nasal Cells of Children

**Authors**: Andres Cardenas, Sheryl L. Rifas-Shiman, Joanne E. Sordillo, Dawn L. DeMeo, Andrea A Baccarelli, Marie-France Hivert, Diane R Gold, Emily Oken

**Table S1.** Demographic characteristics among 547 Project Viva participants with nasal DNA methylation measurements.

| Participant characteristics | N (%) or  mean (SD) |
| --- | --- |
| Sex |  |
| Male | 277 (50.6%) |
| Female | 270 (49.4%) |
| Age (years) | 12.95 (0.66) |
| Race/Ethnicity |  |
| White | 367 (67.1%) |
| Black | 89 (16.3%) |
| Hispanic | 23 (4.2%) |
| Asian | 17 (3.1%) |
| More than one race/Other | 51 (9.3%) |
| DNA methylation telomere length (DNAmTL) Kb | 7.11 (0.38) |
| DNAmTL residuals | 0 (0.38) |
| Skin and Blood Clock Epigenetic age (years) | 15.7 (1.94) |
| Skin and blood clock residuals | 0 (1.77) |

**Table S2.** Differences in %-DNAm of the *ACE2* gene in nasal cells by chronological age in years stratified by sex

| CpG | Females | | |  | Males | | | |
| --- | --- | --- | --- | --- | --- | --- | --- | --- |
|  | %-DNAm  difference | *p* | FDR |  | %-DNAm  difference | *p* | FDR |  |
| cg18458833 | -0.02 | 0.76 | 0.89 |  | -0.11 | 0.43 | 0.89 |  |
| cg21598868 | -0.89 | 0.15 | 0.32 |  | -0.04 | 0.77 | 0.89 |  |
| cg18877734 | -0.34 | 0.06 | 0.18 |  | -0.18 | 0.34 | 0.86 |  |
| cg03536816 | **-2.48** | **0.018** | 0.16 |  | -0.91 | 0.29 | 0.86 |  |
| cg08559914 | 0.01 | 0.96 | 0.96 |  | -0.24 | 0.49 | 0.89 |  |
| cg16734967 | **-1.85** | **0.048** | 0.18 |  | -0.62 | 0.29 | 0.86 |  |
| cg10408040 | -0.1 | 0.66 | 0.83 |  | 0.03 | 0.73 | 0.89 |  |
| cg05241917 | -0.42 | 0.19 | 0.36 |  | -0.12 | 0.62 | 0.89 |  |
| cg20119767 | -0.21 | 0.59 | 0.81 |  | 0.34 | 0.31 | 0.86 |  |
| cg04013915 | -1.48 | 0.07 | 0.18 |  | 0.05 | 0.93 | 0.93 |  |
| cg16716680 | -0.03 | 0.95 | 0.96 |  | 0.17 | 0.75 | 0.89 |  |
| cg25176872 | -0.56 | 0.23 | 0.39 |  | 0.01 | 0.93 | 0.93 |  |
| cg22422976 | **-1.19** | **0.02** | 0.16 |  | -0.04 | 0.75 | 0.89 |  |
| cg05039749 | -0.11 | 0.34 | 0.52 |  | -0.05 | 0.19 | 0.86 |  |
| cg23232263 | -0.96 | 0.04 | 0.18 |  | -0.30 | 0.05 | 0.80 |  |

**Table S3.** Differences in %-DNAm in Asian and more than one race relative to white participants adjusted for age and stratified by sex.

|  | **Females** | | | | | | |  | **Males** | | | | | | |
| --- | --- | --- | --- | --- | --- | --- | --- | --- | --- | --- | --- | --- | --- | --- | --- |
| CpG | **Asian** | | |  | **More than one race** | | |  | **Asian** | | |  | **More than one race** | | |
|  | %-DNAm  difference | *p* | FDR |  | %-DNAm  difference | *p* | FDR |  | %-DNAm  difference | *p* | FDR |  | %-DNAm  difference | *p* | FDR |
| cg18458833 | -0.22 | 0.27 | 0.87 |  | -0.14 | 0.41 | 0.56 |  | 0.33 | 0.48 | 0.96 |  | -0.35 | 0.45 | 0.79 |
| cg21598868 | -0.36 | 0.87 | 0.87 |  | 1.72 | 0.25 | 0.56 |  | 0.20 | 0.66 | 0.96 |  | -0.27 | 0.47 | 0.79 |
| cg18877734 | -0.28 | 0.66 | 0.87 |  | 0.1 | 0.79 | 0.88 |  | 0.10 | 0.89 | 0.96 |  | -0.3 | 0.58 | 0.79 |
| cg03536816 | -1.54 | 0.72 | 0.87 |  | -4.8 | 0.17 | 0.56 |  | 4.30 | 0.03 | 0.38 |  | -0.44 | 0.79 | 0.79 |
| cg08559914 | 0.12 | 0.85 | 0.87 |  | -0.4 | 0.33 | 0.56 |  | 0.58 | 0.49 | 0.96 |  | -0.76 | 0.33 | 0.79 |
| cg16734967 | 1.81 | 0.55 | 0.87 |  | -3.05 | 0.19 | 0.56 |  | 0.87 | 0.65 | 0.96 |  | -0.49 | 0.65 | 0.79 |
| cg10408040 | 0.87 | 0.33 | 0.87 |  | -0.5 | 0.40 | 0.56 |  | -0.47 | 0.31 | 0.96 |  | -0.38 | 0.10 | 0.79 |
| cg05241917 | 0.57 | 0.62 | 0.87 |  | 0.17 | 0.82 | 0.88 |  | 0.33 | 0.64 | 0.96 |  | 0.18 | 0.69 | 0.79 |
| cg20119767 | 1.53 | 0.17 | 0.87 |  | 1.23 | 0.33 | 0.56 |  | -0.05 | 0.96 | 0.96 |  | -0.24 | 0.75 | 0.79 |
| cg04013915 | 1.89 | 0.59 | 0.87 |  | -2.55 | 0.35 | 0.56 |  | 0.75 | 0.55 | 0.96 |  | -0.46 | 0.68 | 0.79 |
| cg16716680 | -0.32 | 0.83 | 0.87 |  | 1.29 | 0.45 | 0.56 |  | -0.13 | 0.95 | 0.96 |  | 0.75 | 0.32 | 0.79 |
| cg25176872 | 1.03 | 0.16 | 0.87 |  | -1.22 | 0.21 | 0.56 |  | 0.02 | 0.96 | 0.96 |  | -0.09 | 0.74 | 0.79 |
| cg22422976 | -0.4 | 0.79 | 0.87 |  | 0.01 | 0.99 | 0.99 |  | 0.31 | 0.42 | 0.96 |  | -0.23 | 0.41 | 0.79 |
| cg05039749 | 0.47 | 0.50 | 0.87 |  | -0.24 | 0.25 | 0.56 |  | 0.01 | 0.93 | 0.96 |  | 0.11 | 0.15 | 0.79 |
| cg23232263 | -0.39 | 0.82 | 0.87 |  | -0.84 | 0.43 | 0.56 |  | 0.16 | 0.64 | 0.96 |  | -0.42 | 0.19 | 0.79 |

**Figure S1.**  Scatter plots, and Pearson’s DNA correlation coefficients between children’s chronological age and DNA methylation aging biomarkers A) Skin & Blood Clock and B) methylation estimator of telomere length (DNAmTL) in nasal cells.


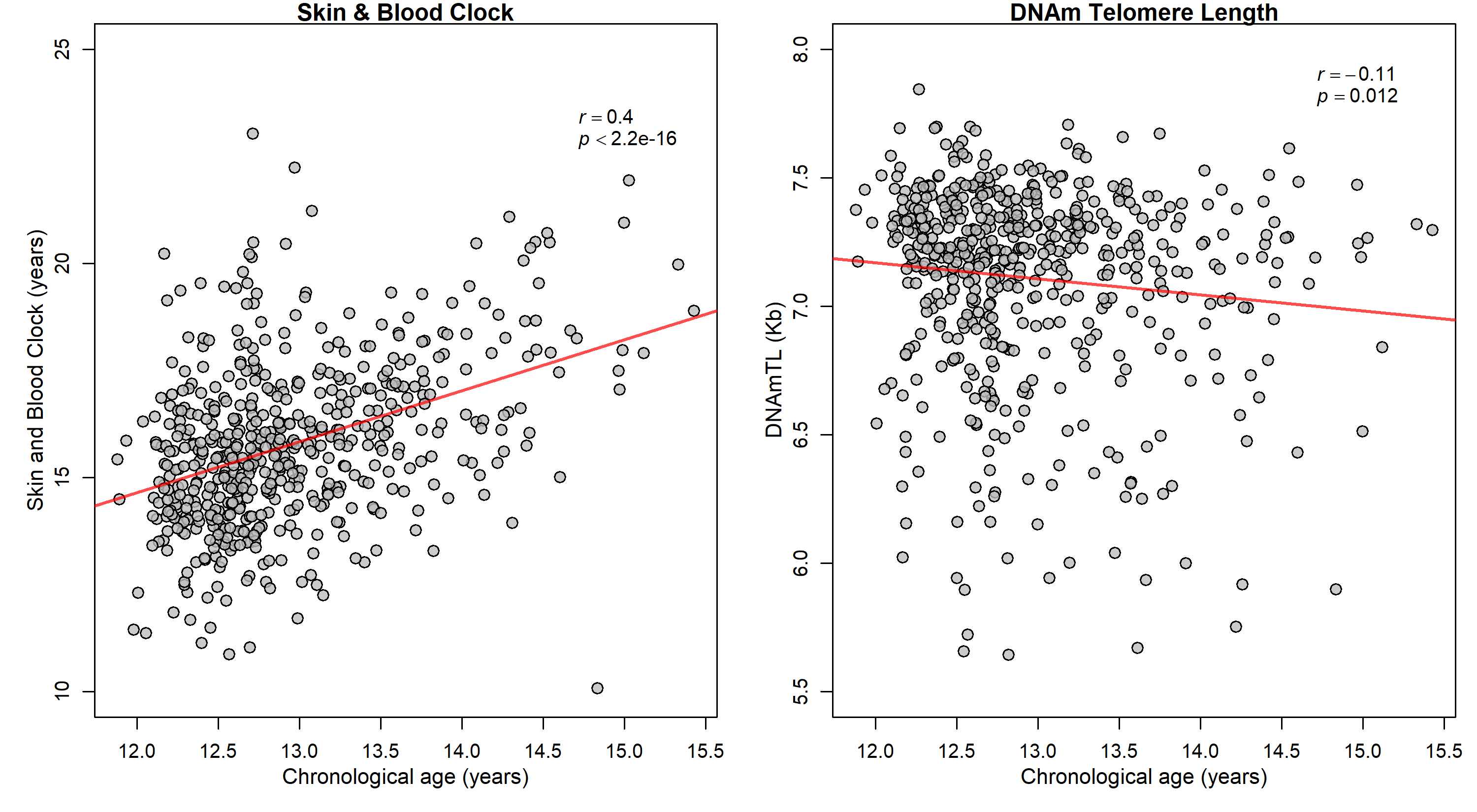


**Figure S2**. Clustering heatmap of DNA methylation in nasal cells for the angiotensin-converting enzyme 2 (*ACE2*) gene among 15 CpG sites


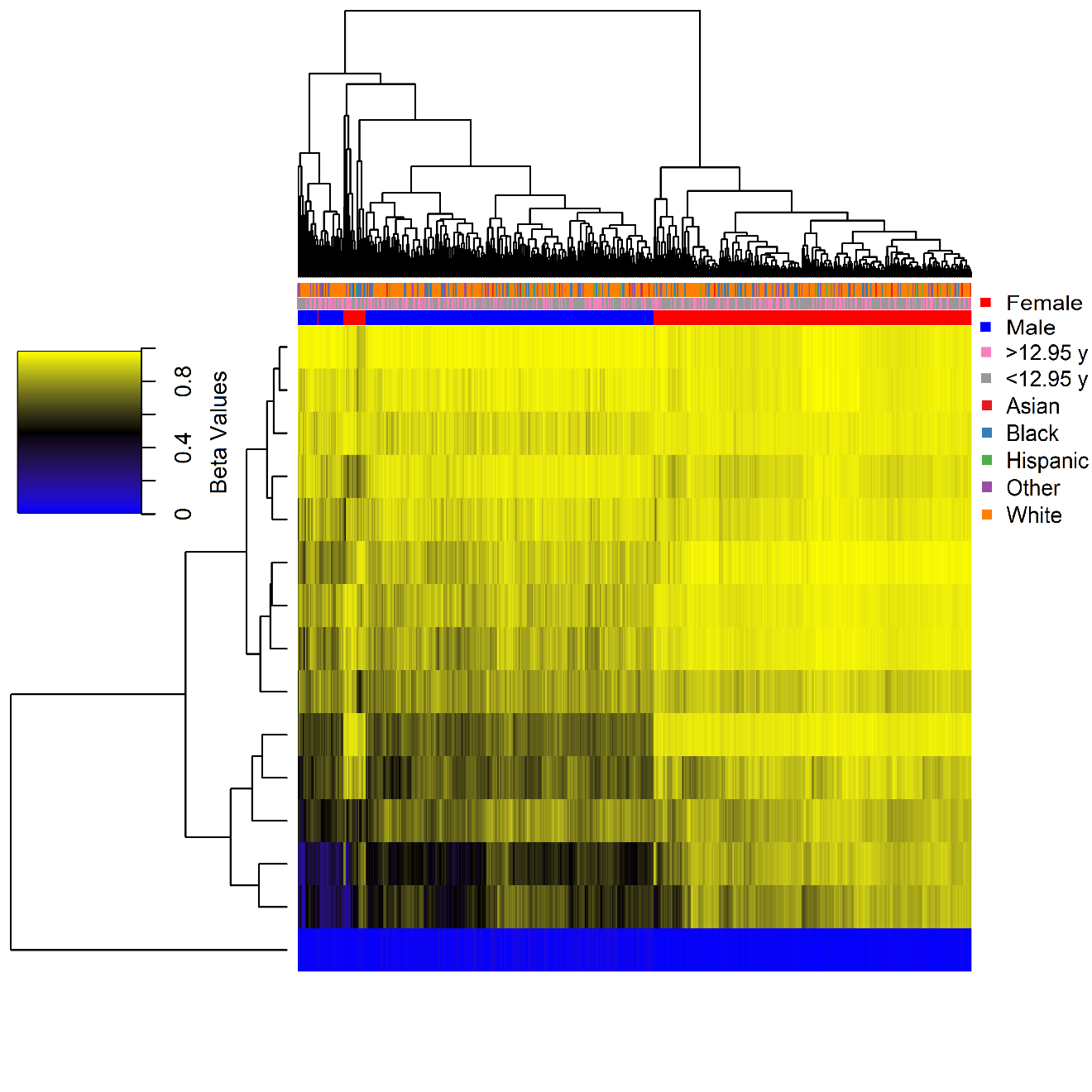

Supplement: Supplementary file 1 — Supplementary Information [file 41598_2021_86494_MOESM1_ESM.docx]
